# Supplementary material for: Dynamic light manipulation via silicon-organic slot metasurfaces
Source: Nat Commun. 2024 Feb 20;15:1557. doi: 10.1038/s41467-024-45544-0 (PMC10879521; doi:10.1038/s41467-024-45544-0)
Supplement: Supplementary file 1 — Supplementary Information [file 41467_2024_45544_MOESM1_ESM.pdf]

# Supporting information: Dynamic Light Manipulation via Silicon-Organic Slot Metasurfaces

Tianzhe Zheng,[1] Yiran Gu,[2] Hyounghan Kwon,[1,3,*] Gregory Roberts,[1,†] and Andrei Faraon[1,3,‡]

[1]*T. J. Watson Laboratory of Applied Physics and Kavli Nanoscience Institute, California Institute of Technology, 1200 E. California Blvd., Pasadena, CA 91125, USA*

[2]*Department of applied physics and material science, California Institute of Technology, 1200 E. California Blvd., Pasadena, CA 91125, USA*

[3]*Department of Electrical Engineering, California Institute of Technology, 1200 E. California Blvd., Pasadena, CA 91125, USA*

* Current address: Center for Quantum Information at Korea Institute of Science and Technology, Address. 5, Hwarang-ro 14-gil, Seongbuk-gu, Seoul, Republic of korea

† Current address: Tech4Health Institute, New York University Langone Health, New York, NY 10016, USA

‡ Corresponding author: A.F.: faraon@caltech.edu

## SUPPLEMENTARY NOTE 1: CREATION OF THE SLOT MODE RESONANCE

In this section, the principle of slot mode resonance creation is discussed. The idea comes from previous research on high-Q resonances[1, 2]. In integrated photonics, a slot waveguide is used for light propagation because its momentum is larger than the momentum of free-space light. This bounded-state property guarantees zero radiation loss in the ideal slot waveguide. However, the subtle periodic perturbation will compensate for the momentum mismatch and enable the radiation into the free space. For example, by targeting $k_y = 0$, we can couple the slot waveguide to the z-incident light, as illustrated in Fig. 1 of the main manuscript. Specifically, the slot mode has a dispersion relationship shown in Fig. **S1**. We wrap the dispersion curve within the range of $\pi/p$. Under this operation, the curve reaches $k_y = 0$ at a specific wavelength of $\lambda = 1591.6nm$. In other words, under the perturbation with period $p$, $k_y$ will be wrapped to 0 and thus couples to the normal incident light.

We also numerically confirm the existence of the resonance in Fig. **S2** and the properties of perturbations. Simulations reveal three effects depending on the notch size: the blue shift from the non-perturbed resonance wavelength, the quality factor, and the modulation amplitude. Firstly, the geometrical perturbation by creating notches leads to the blue shift according to the first order perturbation theory[3]. Secondly, the quality factor increases as the perturbation disappears. This indicates that the notch size controls the radiative loss of the slot mode, and it is the origin of the resonance. Thirdly, shown in Fig. 4 of the main manuscript, the smaller the notch size, the smaller the modulation amplitude, which is the result of the decreasing coupling rate. In conclusion, the notch size and notch period are two powerful knobs that enable resonance creation at a large range of wavelengths and with arbitrary quality factors.

## SUPPLEMENTARY NOTE 2: CALCULATION OF THE OVERLAPPING FACTOR

The interaction strength between the polymer and optical mode has previously been investigated, including that between polymer and guided mode[4] and that between polymer and non-perturbed slot mode[5]. The calculation formula of the overlapping factor ($\Gamma_c$) has been proposed in equation (24) in the supplementary material of reference[4], under the quantum picture. Here, we will provide another derivation of the same equation from the cavity perturbation theory[3].

The basic picture of the electro-optic-induced resonance shift could be decomposed in several steps. First, the mode frequency shift is caused by the local dielectric tensor perturbation $\Delta\overline{\overline{\epsilon}}$. Second, the local anisotropic dielectric tensor perturbation is generated by tuning the electric field $\mathbf{E}_{\text{ext}}$ under non-negligible electro-optic coefficient $r_{33}$. Third, the direction and the amplitude of $r_{33}$ depend on the poling electric field $\mathbf{E_p}$. Note that the bold variables $\mathbf{E}_{\text{ext}}$, $\mathbf{E_p}$ means they are vectors, and all these variables are dependent on the locations. We will walk through the calculation step by step.

In the first step, the $i$th mode frequency shift $\Delta\omega_i$ when we have a dielectric tensor perturbation could be easily calculated by perturbation theory [3]:

$$\frac{\Delta\omega_i}{\omega_i} = -\frac{1}{2}\frac{< \mathbf{E_i}, \Delta\overline{\overline{\epsilon}}\mathbf{E_i} >}{< \mathbf{E_i}, \overline{\overline{\epsilon}}\mathbf{E_i} >} \tag{S1}$$

Here, $\mathbf{E_i}$ is the vector of $i$th mode field, $\overline{\overline{\epsilon}}$ and $\overline{\overline{\Delta\epsilon}}$ are the dielectric tensor of the material and its perturbation. The inner product is the integral across the region of one period in both $x$ and $y$ directions, and infinity in $z$ direction:

$$\int_{-\infty}^{\infty}\int_{-p/2}^{p/2}\int_{-w_p/2}^{w_p/2} \mathbf{E_1}^{\dagger}\overline{\overline{\epsilon}}\mathbf{E_2}\mathrm{d}x\mathrm{d}y\mathrm{d}z :=< \mathbf{E_1}, \overline{\overline{\epsilon}}\mathbf{E_2} > \tag{S2}$$

$\mathbf{E_1}^{\dagger}$ means the conjugate transpose of the vector. In the second step, the “local" perturbation of the dielectric tensor $\Delta\overline{\overline{\epsilon}}$ is dependent on “local" electro-optic coefficient $r_{33}$. The definition of the electro-optic coefficient is by relative impermeability $\overline{\overline{\eta}} = (\frac{\overline{\overline{\epsilon}}}{\epsilon_0})^{-1}$, and here we are only interested in non-negligible coefficient $r_{33}$[6]:

$$\Delta\eta'_{33} = r_{33}|\mathbf{E}_{\text{ext}}| = r_{33}E_{ext,z'} \tag{S3}$$

We define a local coordinate system $(x', y', z')$, where $z'$ axis is the same direction as the local poling electric field $\mathbf{E_p}$. $\Delta\eta'_{33}$ means that the variable is expressed in coordinate system $(x', y', z')$. Since the poling field and the tuning field use the same electrodes, thus under this local coordinate system the relationship between $\mathbf{E}_{\mathrm{ext}}$ and $\Delta\bar{\bar{\eta}}$ is simpler (only $r_{33} \neq 0$ and $\mathbf{E}_{\mathrm{ext}} = (0, 0, E_{\mathrm{ext,z'}})^T$), shown in Equation S3. If we write the equation in tensor form, the result is:

$$\overline{\overline{\Delta\eta'}} = \frac{\mathbf{E}'_{\mathrm{ext}}\mathbf{E}'_{\mathrm{ext}}{}^{\dagger}}{|\mathbf{E}'_{\mathrm{ext}}|} \tag{S4}$$

The transformation matrix between the local axis base vectors $\mathbf{e'_i}$ and the global axis base vectors $\mathbf{e_i}$ are defined by Q:

$$\mathbf{e_i} = Q_{ij}\mathbf{e'_j} \tag{S5}$$

Then the dielectric constant perturbation could be expressed:

$$\Delta\bar{\bar{\epsilon}} = -\frac{1}{\epsilon_0}\bar{\bar{\epsilon}} \cdot \overline{\overline{\Delta\eta}} \cdot \bar{\bar{\epsilon}} = -\frac{1}{\epsilon_0}\bar{\bar{\epsilon}} \cdot Q\overline{\overline{\Delta\eta'}}Q^T \cdot \bar{\bar{\epsilon}} \tag{S6}$$

$$= -\frac{1}{\epsilon_0} r_{33}\bar{\bar{\epsilon}} \cdot Q\frac{\mathbf{E}'_{\mathrm{ext}}\mathbf{E}'_{\mathrm{ext}}{}^{\dagger}}{|\mathbf{E}'_{\mathrm{ext}}|}Q^T \cdot \bar{\bar{\epsilon}} \tag{S7}$$

$$= -\frac{1}{\epsilon_0} r_{33}\bar{\bar{\epsilon}} \cdot \frac{\mathbf{E}_{\mathrm{ext}}\mathbf{E}_{\mathrm{ext}}{}^{\dagger}}{|\mathbf{E}_{\mathrm{ext}}|} \cdot \bar{\bar{\epsilon}} \tag{S8}$$

In the third step, we need to connect the local variant $r_{33}$ with the poling and the tuning voltage. We define the poling efficiency $k_p$:

$$k_p = r_{33}/|\mathbf{E_p}| \tag{S9}$$

Since we are using the same electrode for poling and tuning, $\mathbf{E_p}$ and $\mathbf{E}_{\mathrm{ext}}$ follow the same distribution. For simplicity, we could define $\mathbf{E_p} = \alpha\mathbf{E}_{\mathrm{ext}}$. $k_p$ is a constant within the OEO region, and outside OEO region $k_p = 0$. $\alpha$ is number that is not dependent on the locations. Therefore, the inner product in equation S2 could be expressed as

$$< \mathbf{E_i}, \Delta\bar{\bar{\epsilon}}\mathbf{E_i} > = -\frac{1}{\epsilon_0} < \mathbf{E_i}, \bar{\bar{\epsilon}} \cdot k_p\alpha\mathbf{E}_{\mathrm{ext}}\mathbf{E}^{\dagger}_{\mathbf{ext}} \cdot \bar{\bar{\epsilon}}\mathbf{E_i} > \tag{S10}$$

$$= -\frac{k_p\alpha}{\epsilon_0} < \mathbf{E}^{\dagger}_{\mathbf{ext}}\bar{\bar{\epsilon}} \cdot \mathbf{E_i}, \mathbf{E}^{\dagger}_{\mathbf{ext}}\bar{\bar{\epsilon}} \cdot \mathbf{E_i} >_{\mathrm{OEO}} \tag{S11}$$

We define the inner product $< \cdot, \cdot >_{\mathrm{OEO}}$ when the integral only includes the region of OEO

materials. The shift of the frequency due to electro-optic field is

$$\frac{\Delta\omega_i}{\omega_i} = \frac{k_p \alpha}{2\epsilon_0} \frac{< \mathbf{E}_{\mathbf{ext}}^{\dagger} \overline{\overline{\epsilon}} \cdot \mathbf{E_i}, \mathbf{E}_{\mathbf{ext}}^{\dagger} \overline{\overline{\epsilon}} \cdot \mathbf{E_i} >_{\mathrm{OEO}}}{< \mathbf{E_i}, \overline{\overline{\epsilon}} \mathbf{E_i} >} \tag{S12}$$

$$= \frac{k_p \alpha}{2\epsilon_0} \frac{\iiint_{\mathrm{poly}} \epsilon^2 |\mathbf{E}_{\mathbf{ext}}^{\mathbf{T}} \mathbf{E}_i|^2 \mathrm{d}x\mathrm{d}y\mathrm{d}z}{\iiint_{\mathrm{everywhere}} \epsilon |\mathbf{E}_i|^2 \mathrm{d}x\mathrm{d}y\mathrm{d}z} \tag{S13}$$

We made two assumptions in the equation S13. First, although we consider the anisotropic perturbation of the dielectric tensor, we assume that the dielectric tensor $\overline{\overline{\epsilon}}$ is isotropic. Thus we can use a scalar $\epsilon$ instead of tensor. Second, we assume that $\mathbf{E}_{\mathrm{ext}}$ is a real vector as the tuning source frequency is much lower compared to the optical oscillation frequency.

If we approximate the average poling field as $|\overline{\mathbf{E_p}}| = V_p/w_g$, then we can estimate the average $\overline{r_{33}} = k_p|\overline{\mathbf{E_p}}| = k_p V_p/w_g$. Given tuning voltage $V_{\mathrm{ext}}$, we could also get $\alpha = \frac{V_p}{V_{\mathrm{ext}}}$. The definition of the overlap factor is

$$\frac{\Delta\omega}{\omega} = \frac{1}{2}\frac{\epsilon}{\epsilon_0}\overline{r_{33}}\Gamma_c \frac{V_{\mathrm{ext}}}{w_g} \tag{S14}$$

where $V$ is the voltage, $w_g$ is the slot gap width. Incorporate all these equations we can get the expressions for overlapping factor,

$$\Gamma_c = \frac{w_g^2}{V_{\mathrm{ext}}^2} \frac{\iiint_{\mathrm{poly}} \epsilon |\mathbf{E}_{\mathbf{ext}}^{\mathbf{T}} \mathbf{E_i}|^2 \mathrm{d}x\mathrm{d}y\mathrm{d}z}{\iiint_{\mathrm{everywhere}} \epsilon |\mathbf{E}_i|^2 \mathrm{d}x\mathrm{d}y\mathrm{d}z} \tag{S15}$$

$\mathbf{E}_{\mathrm{ext}}$ has a linear relationship with $V_{\mathrm{ext}}/w_g$, so the overlapping factor is not relevant to the externally applied voltage.

Upon applying the above formula for the three modes discussed in the main text and the Au strip-integrated device, we record results in Table S1. The overlap factor decreases slightly with

| Modes | I. | II. | III. | I.(with gold) |
|---|---|---|---|---|
| $\Gamma_c$ | 0.156 | 0.017 | 0.015 | 0.127 |

**Table S1 The overlap factor $\Gamma_c$ of the optical modes discussed in Fig.2 in the main manuscript and Fig. S3.**

the integration of Au strips due to the existence of metal absorption. Also, compared to the guided modes II and III, the slot mode achieves around one order of magnitude higher overlapping factor.

## SUPPLEMENTARY NOTE 3: EFFECT OF ADDITIONAL METAL STRIP ON THE LOSS OF THE SLOT MODE RESONANCE

To ensure a minimal metallic loss, metal strips are integrated at the center of the silicon rail and have a width of $100nm$. For $850nm$-wide slabs, metal strips have limited effect on the overlapping factor $\Gamma_c$, the resonance shift, and the overall absorption. Firstly, $\Gamma_c$ decreases by 0.03 upon this insertion, shown in Tab. **S1**. Secondly, informed from the simulated reflection spectra in Fig. **S3**, the resonance shift from $n = 1.85$ to $n = 1.87$ is $2.71nm$. Thus, the extra metal strips hardly affect the sensitivity of the slot mode with respect to the electro-optic effect of the polymer. Finally, the calculated quality factors of the resonances with and without metal strips are 1864 and 1979 (Fig. 2f in the main manuscript), respectively. Both quality factors are just slightly above our experimental realization, and the effect of the metal is minimal. In conclusion, the metal strip will only have minimal effect on the resonance property and the tuning performance.

## SUPPLEMENTARY NOTE 4: THE SIMPLIFIED CIRCUIT MODEL AND THE RF MEASUREMENT FOR THE BANDWIDTH ESTIMATION

In this section, we further discuss the simplified circuit model which has been used in Fig. 6b in the main manuscript. The model parameters are chosen as described in the following paragraph. To determine the capacitance in the circuit and determine the primary limiting factor of the bandwidth in our device, we conduct electric transmission tests on different structures. The outcomes and the modeling circuits for each structure are shown in Fig. **S4**, and the illustrations of experimental setups are shown in Fig. **S5**.

First, we assess the bandwidth of the PCB board. The schematic diagram of the measurement setup is shown in Fig. **S5**a, and the result is represented by the purple curve in Fig. **S4**a. It is clear that signals below 20MHz remain unaffected. The bandwidth of the PCB board is significantly larger than all the other measurements; therefore, we do not take into account the effect of the PCB on the signal bandwidth.

Next, we measure the transmission of signals $V_{\text{out}}$ through the PCB and an coplanar waveguide (CPW). Despite continuing to use a large electrode pad for wire bonding, the coplanar waveguide could significantly reduce the transmission loss through the waveguide. The gold electrode waveguide is situated on top of a thin film undoped silicon layer with a fused silica substrate[4]. The schematic diagram of the measurement setup can be seen in Fig. **S5**b. The measured data exhibits 3dB attenuation at 21MHz.We model the circuit as a RC circuit shown in Fig. **S4**b. The calculation of $V_{\text{load}}$ is:

$$V_{\text{load}} = |\frac{Z_{\text{load}}}{R + Z_{\text{load}}}| \qquad \textbf{(S16)}$$

where $Z_{\text{load}} = \frac{1}{j2\pi f C_{\text{circ}}}$. The calculated capacitive load $C_{\text{circ}} = 262.5\text{pF}$.

Third, we measure the transmission spectra of signals through the PCB and the optimized RF waveguide on the SOI wafer in air (Measurement data, circuit and diagram II in Fig. **S4**a-b and Fig. **S5**b). The SOI wafer introduces additional capacitance $C_{\text{SOI'}}$ between gold electrodes and the doped silicon substrate. Therefore in case II, $Z_{\text{load}} = \frac{1}{j2\pi f (C_{\text{circ}}+C_{\text{SOI'}})}$. Based on the measurement, the 3dB cutoff frequency is 6.8MHz. Therefore, we can estimate the capacitance of the SOI wafer in this case as $C_{\text{SOI'}} = 548.24\text{pF}$. As a sanity check, if we treat the SOI wafer capacitance as the

parallel plate, then the capacitance $C_{\mathrm{SOI'}}$ could be expressed as:

$$C_{\mathrm{SOI'}} = \frac{\epsilon_0 \epsilon_{\mathrm{SiO_2}} S_{\mathrm{SOI'}}}{2 h_{\mathrm{SiO_2}}} \tag{S17}$$

$\epsilon_0$ is the vacuum permittivity. We can solve the area of the whole waveguide $S_{\mathrm{SOI'}} = 9.5mm^2$. It is similar to the area of the electrode design since there are two large pads for wire bonding.

As our main device doesn't incorporate the CPW, the impedance unmatching through the waveguide will impact the load voltage $V_{\mathrm{load}}$. To identify other possible parasitic capacitances in the circuit, we measure the loading voltage signal $V_{\mathrm{load}}$ with another test chip with a smaller electrode area (Measurement data, circuit and diagram III in Fig. S4a-b and Fig. S5b). The measurement 3dB cutoff frequency is 4MHz. In this device, we assume that $Z_{\mathrm{load}} = C_{\mathrm{circ}} + C_{\mathrm{SOI'}} + C_{\mathrm{other}}$, which implies that the contribution of all other factors except for the original contribution in case I and SOI capacitance contribution in case II could be modeled as a capacitor $C_{\mathrm{other}}$. We also assume that the area of the electrode is the same as in case II. The result $C_{\mathrm{other}} = 567.5$pF. As such, we define the external capacitance of our device in optical measurement $C_{\mathrm{load}} = C_{\mathrm{circ}} + C_{\mathrm{other}} = 813.5$pF. The substrate contribution of the device in the main figure is determined by the electrode area $S_{\mathrm{SOI}} = 16mm^2$ in the device $C_{\mathrm{SOI}}/2 = 920.82$pF using equation S17(Since in Fig. 6 of the main manuscript the capacitance between the electrode and substrate is labeled as $C_{\mathrm{SOI}}$). Based on all these discussions, the final model shown in Fig. 6b of the main manuscript considers two major capacitances, $C_{\mathrm{load}}$ and $C_{\mathrm{SOI}}$, to capture the effect of the parasitic capacitance along the circuit and the substrate.

Within the device, we treat the electric path across the slot as two gold nanobars $R_{\mathrm{Au}}$, two resistive silicon nanobars $R_{\mathrm{Si}}$, a resistive component $R_{\mathrm{OEO}}$ and a capacitive component $C_{\mathrm{OEO}}$. $R_{\mathrm{OEO}}$ and $C_{\mathrm{OEO}}$ are parallel connected to each other and series connected to $R_{\mathrm{Si}}$ and $R_{\mathrm{Au}}$, as shown in Fig. 6 of the main manuscript. The resistance of the gold nanobar can be modeled as $R_{\mathrm{Au}} = \frac{\rho_{\mathrm{Au}} w_{\mathrm{Au}}}{h_{\mathrm{Au}} l_{\mathrm{Au}}} = 338\Omega$, where $\rho_{\mathrm{Au}} = 2.2 \times 10^{-6}\Omega \cdot cm$, $l_{\mathrm{Au}} = 100\mu m$, $h_{\mathrm{Au}} = 65nm$. Thanks to the extruded gold electrode, the resistance of the silicon bar can be modeled as $R_{\mathrm{Si}} = \frac{\rho_{\mathrm{Si}} w_{\mathrm{Si}}}{h_{\mathrm{Si}} l_{\mathrm{Si}}} = 1.17k\Omega$, where $\rho_{\mathrm{Si}} = 10\Omega \cdot cm$, $l_{\mathrm{Si}} = 100\mu m$, $w_{\mathrm{Si}} = 350nm$, $h_{\mathrm{Si}} = 300nm$. The resistance of the OEO material is modelled as $R_{\mathrm{OEO}} = \frac{\rho_{\mathrm{OEO}} w_{\mathrm{OEO}}}{h_{\mathrm{OEO}} l_{\mathrm{OEO}}} = 200G\Omega$, where $\rho_{\mathrm{OEO}} = 6 \times 10^9\Omega \cdot cm$, $l_{\mathrm{OEO}} = 100\mu m$,$h_{\mathrm{OEO}} = 300nm$. The capacitance of the polymer is modeled approximately as a parallel capacitor, thus $C_{\mathrm{OEO}} = \frac{\epsilon_0 \epsilon_{\mathrm{OEO}} h_{\mathrm{OEO}} l_{\mathrm{OEO}}}{w_{\mathrm{OEO}}} = 21.25$fF, where $\epsilon_{\mathrm{OEO}} = 8$. The impedance of one slot is then $Z_{\mathrm{single}} = 2R_{\mathrm{Au}} + 2R_{\mathrm{Si}} + Z_{\mathrm{OEO}}$, where $Z_{\mathrm{OEO}} = \frac{R_{\mathrm{OEO}}}{1+j2\pi f C_{\mathrm{OEO}}}$. Thus, the total impedance of

the device is $Z_{\text{dev}} = Z_{\text{single}}/N_{\text{slot}}$ where $N_{\text{slot}} = 90$.

Finally, incorporating all previous calculations we can get:

$$V_{\text{OEO}} = V \frac{Z_{\text{dev}}}{Z_{\text{total}}} \frac{Z_{\text{OEO}}}{Z_{\text{single}}} \tag{S18}$$

The calculated result is shown in Fig. 6a in the main manuscript.

## SUPPLEMENTARY NOTE 5: DISCUSSION ON THE STRATEGIES OF BANDWIDTH IMPROVEMENT

Based on the discussion in supplementary note 4, we have identified the source factor of the bandwidth limitation. To increase the bandwidth of the device, we suggest the following strategies:

- Use doped silicon on fused silica wafer to remove $C_{\mathrm{SOI}}$. This type of wafer will require the development of a specialized fabrication process, but it could be potentially constructed by ion implantation and annealing. This improvement could be supported by experiments I and II, as the replacement of the wafer will lead to the increase of the bandwidth in experiment I.
- The design and implementation of a microwave coplanar waveguide, combined with the use of a probe instead of wire bonding and meticulous removal of possible parasitic capacitance in the circuit, could effectively minimize $C_{\mathrm{load}}$, and it has been shown in ref. [7]. We expect that in ideal case the bandwidth should only be restricted by $R_{\mathrm{Au}}$,$R_{\mathrm{Si}}$,$C_{\mathrm{OEO}}$ and $R_{\mathrm{OEO}}$.
- The careful adjustment of doping levels, device length, and device geometry to tailor them toward achieving GHz bandwidth presents another feasible avenue for optimization. As shown in supplementary note 4, the bandwidth limitation by $R_{\mathrm{Au}}$,$R_{\mathrm{Si}}$,$C_{\mathrm{OEO}}$ and $R_{\mathrm{OEO}}$ is approximately $1/(2\pi(R_{\mathrm{Si}}+R_{\mathrm{Au}})C_{\mathrm{OEO}}) = 4.8\mathrm{GHz}$. The bandwidth could be further improved if we reduce the device length or increase the doping levels.

Therefore, we believe the silicon-organic electro-optic modulation device based on slot waveguide could achieve GHz bandwidth.

## SUPPLEMENTARY NOTE 6: FABRICATION AND POLING WORKFLOWS, MEASUREMENT SETUP

The detailed fabrication workflow is shown in Fig. **S6**.

The measurement setup , which has been discussed in detail in our previous report[8], is shown in Figure **S7**.

## SUPPLEMENTARY NOTE 7: DEVICE PARAMETER DETAILS

We list the parameter details of the devices simulated or fabricated in the main figures. The symbols for the geometrical parameters are labeled in Fig. S8. All devices are fabricated using an SOI wafer with a 300-nm device layer and a 300-nm BOX layer. For fabricated devices, the listed parameters are the design layout parameters with estimated geometry shifts due to fabrication. We estimate the geometry shift by the SEM image of other devices on the same chip.

| Device | $p$ | $w_g$ | $w_s$ | $d$ | $l$ | $w_m$ |
|---|---|---|---|---|---|---|
| Figure 2 | 740 | 90 | 850 | 50 | 120 | N/A |
| Figure 4 | Varied | 100 | 830 | Varied | Varied | 100 |
| Figure 5a | 720 | 90 | 840 | 60 | 130 | 100 |
| Figure 5b | 900 | 80 | 650 | 55 | 133 | 100 |
| Figure 5c | 880 | 80 | 650 | 55 | 133 | 100 |

**Table S2** Device parameters used for the plot in main figures (unit: nm). N/A: not applicable. Varied: the certain parameter is a variable in the main figures.

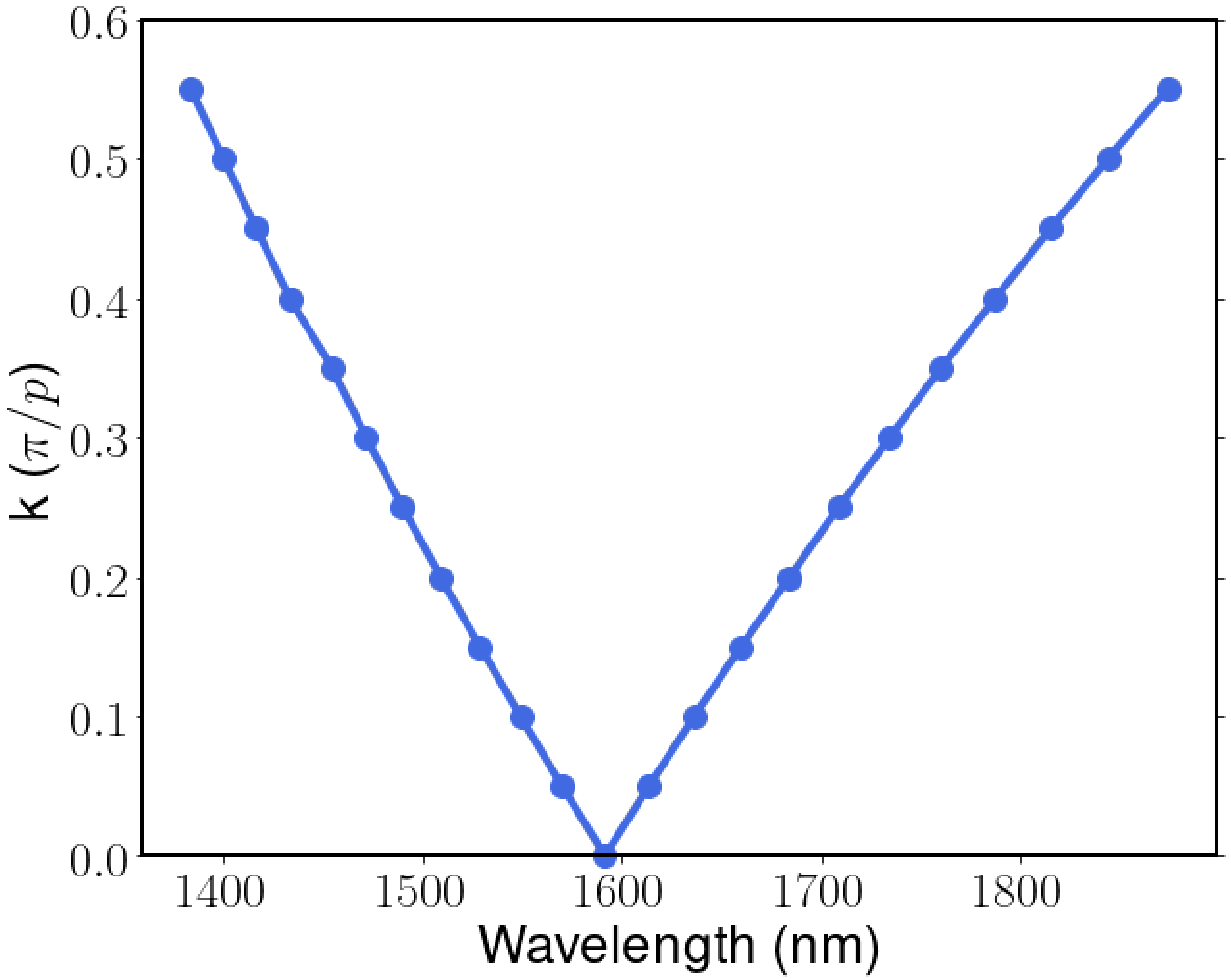


**Figure S1 Band diagram of the slot mode without perturbation**. The calculated slot mode wavelength for different in-slot momentum $k_y$ along the slot. The in-slot momentum is wrapped under the unit of $\pi/p$ where $p = 740nm$. At $\lambda = 1591.6nm$, $k_y = 0$, indicating that the perturbation in the $740nm$ period will open the resonance at this wavelength.

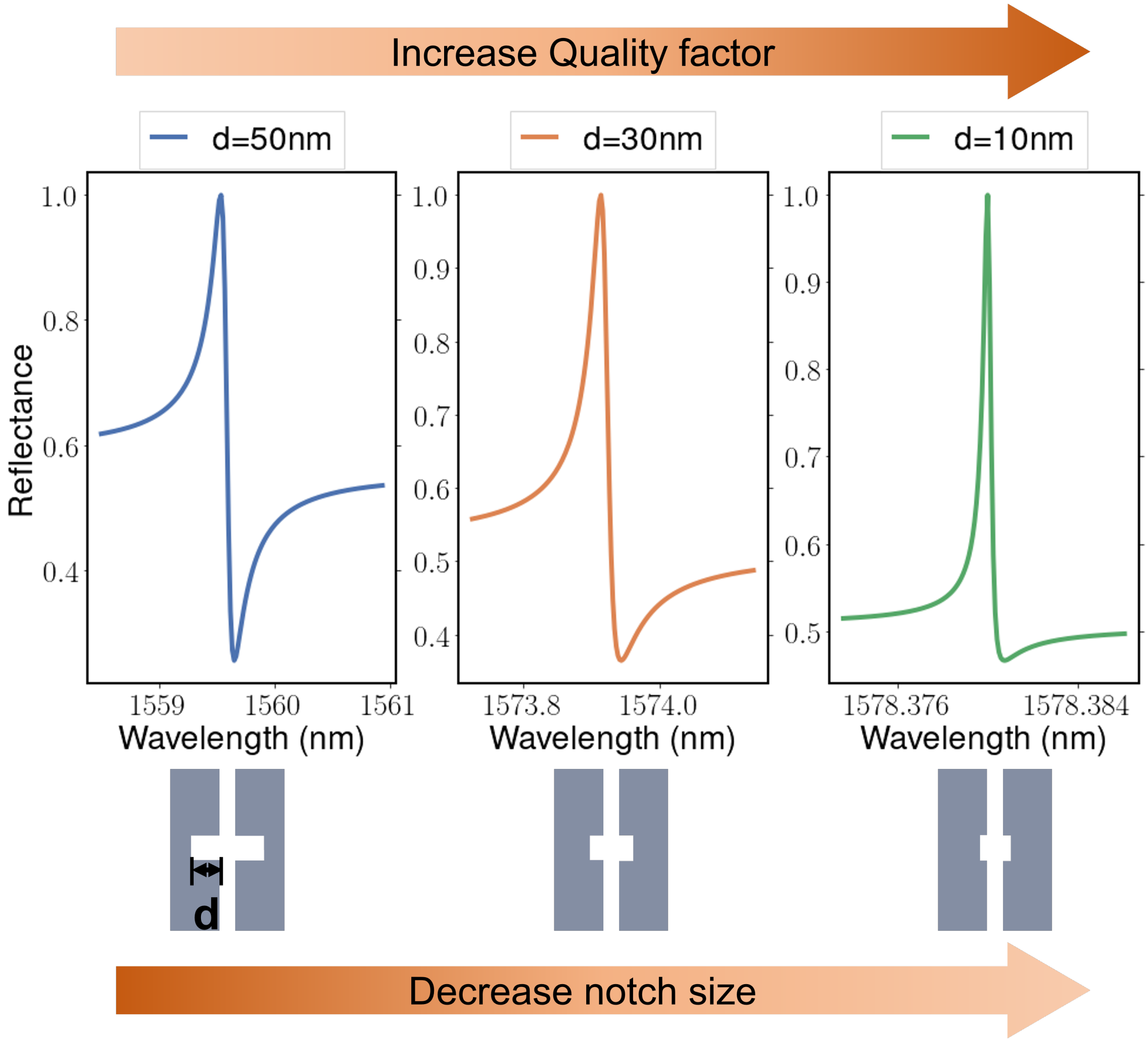


**Figure S2 Creation of the slot mode resonance**. From left to right: the reflection spectra when decreasing the width of the notch $d$ from $50nm$ to $10nm$. The simulated structure is the same as shown in Fig. 2 of the main manuscript.

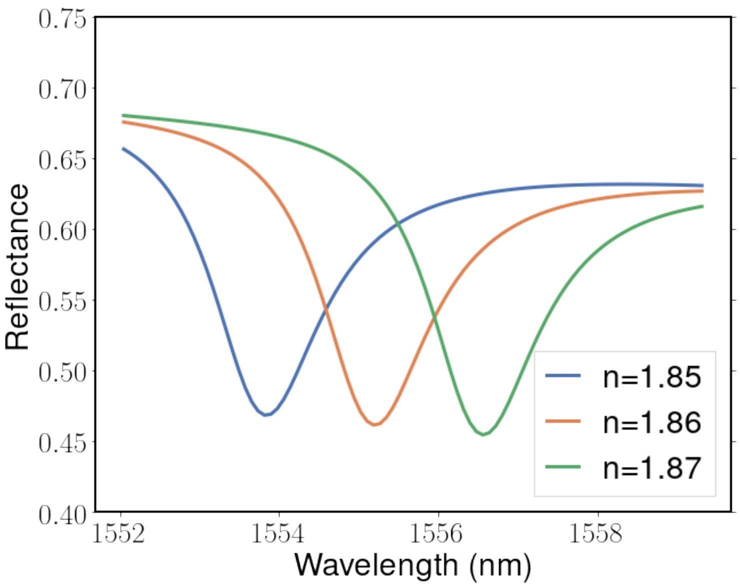


**Figure S3 Numerical calculation of the reflection spectra for the device with metal strips** The spectra with different refractive indexes in the active region are plotted. The device structure is the same as shown in Fig. 2 of the main manuscript but with the metal strip. The shift between $n = 1.85$ and $n = 1.87$ is 2.71nm.

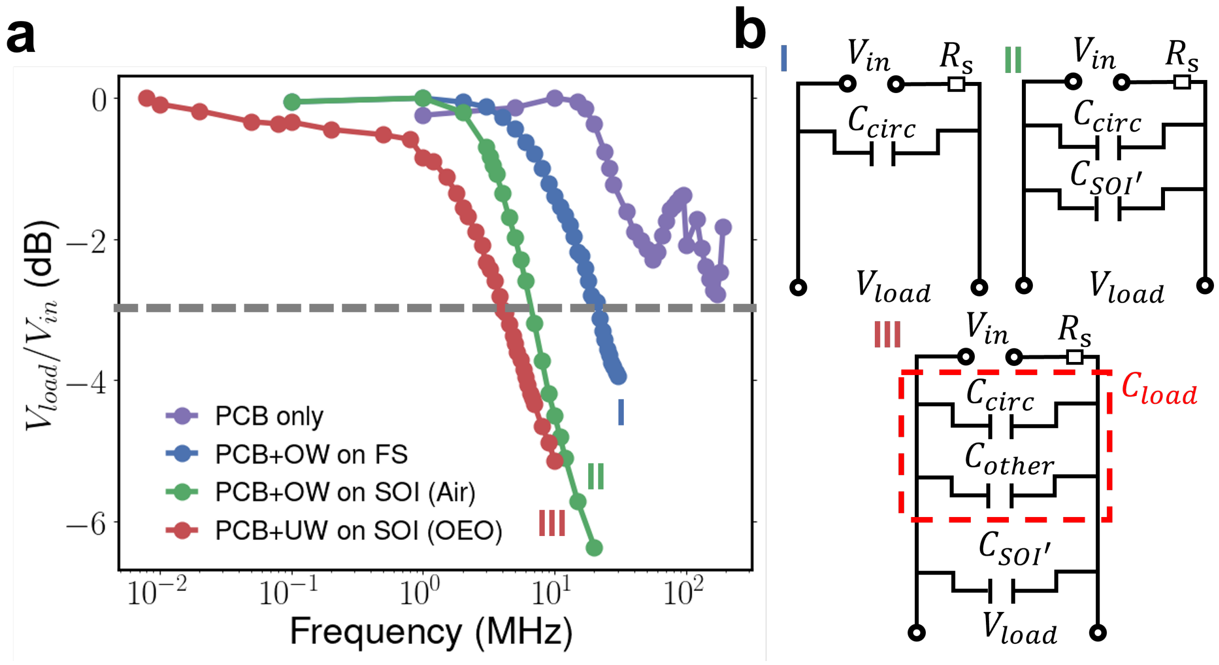


**Figure S4 Electrical tests for the circuit on other device structures.** The detected signal is the electric transmission signal ($V_{\text{load}}$) that passes through the electrode layout instead of optical modulation signal. a. measurement transmission $V_{\text{out}}/V_{\text{in}}$ as a function of frequency. b. The equivalent circuit model for different structures. Purple curve : the signal only passes the PCB board. Blue curve(I): the signal passes the PCB board and an optimized waveguide (OW). The OW achieves impedance matching in most propagation region. The connections between PCB and waveguides are wire bonded. The substrate is replaced as fused silica. The 3dB bandwidth is 21MHz. $R_s$: source resistance. $C_{\text{circ}}$: the additional capacitance within PCB-bonding wire-waveguide circuit. Green curve(II): same setting as I but the substrate is SOI substrate. The waveguide is exposed to an air environment. The 3dB bandwidth is 6.8MHz. $C_{\text{SOI}'}$: the additional capacitance for optimized waveguide due to SOI substrate. Red curve(III): The device is coated with OEO material, and the waveguide is the same as the device in Fig. 6 of the main manuscript with a smaller wire bonding pad. The 3dB bandwidth is 4MHz. $C_{\text{other}}$: the additional capacitance due to impedance unmatching.

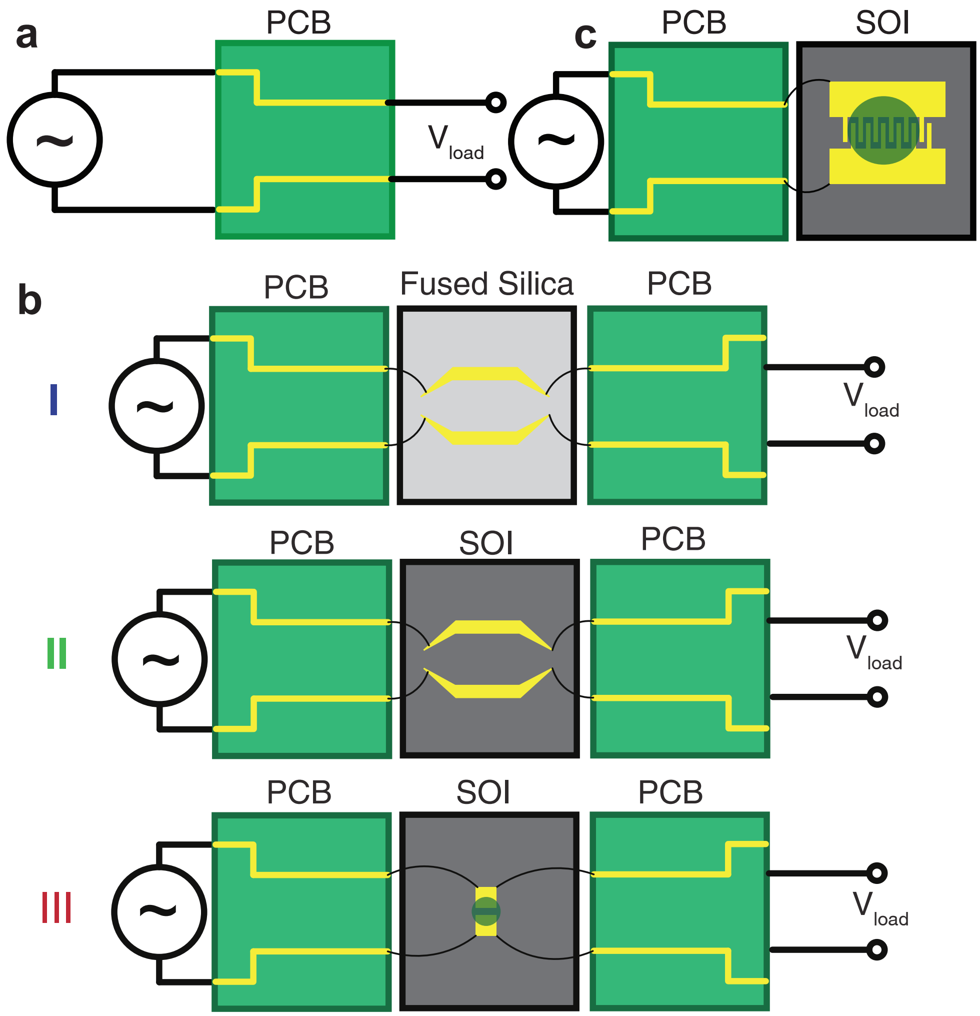


**Figure S5 Schematic diagrams of the AC measurements**. **a**. The diagram of the AC measurement for PCB speed. It corresponds to the PCB only measurement data in Fig. S4a. **b.** The diagrams of the AC measurement for case I,II,III in Fig. S4. **c**. The diagrams of the AC measurement for Fig. 6 of the main manuscript.

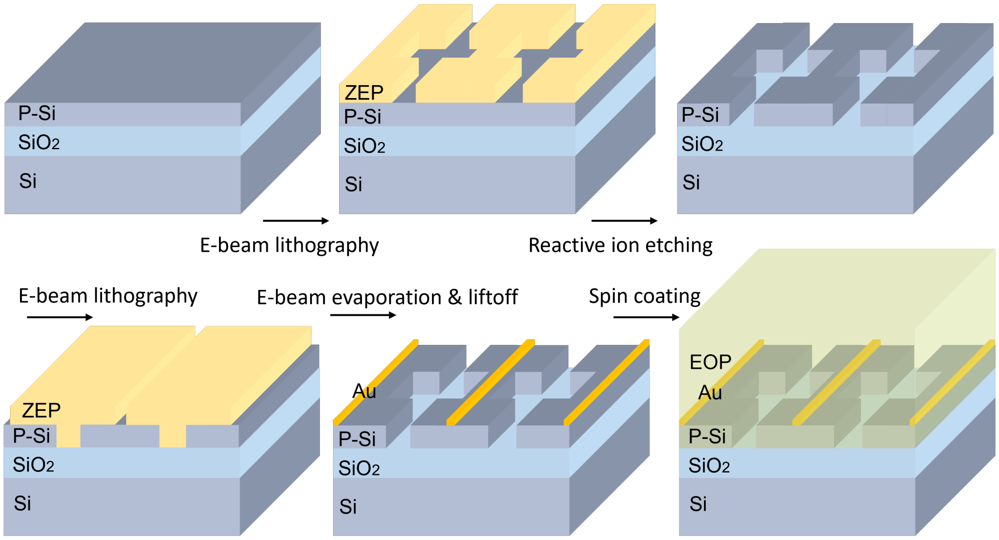


**Figure S6 Fabrication workflow.** Top row: the fabrication of the slot by E-beam lithography and ICP-RIE etching. Bottom row: the fabrication of the electrode by metal liftoff and the coating of the polymer.

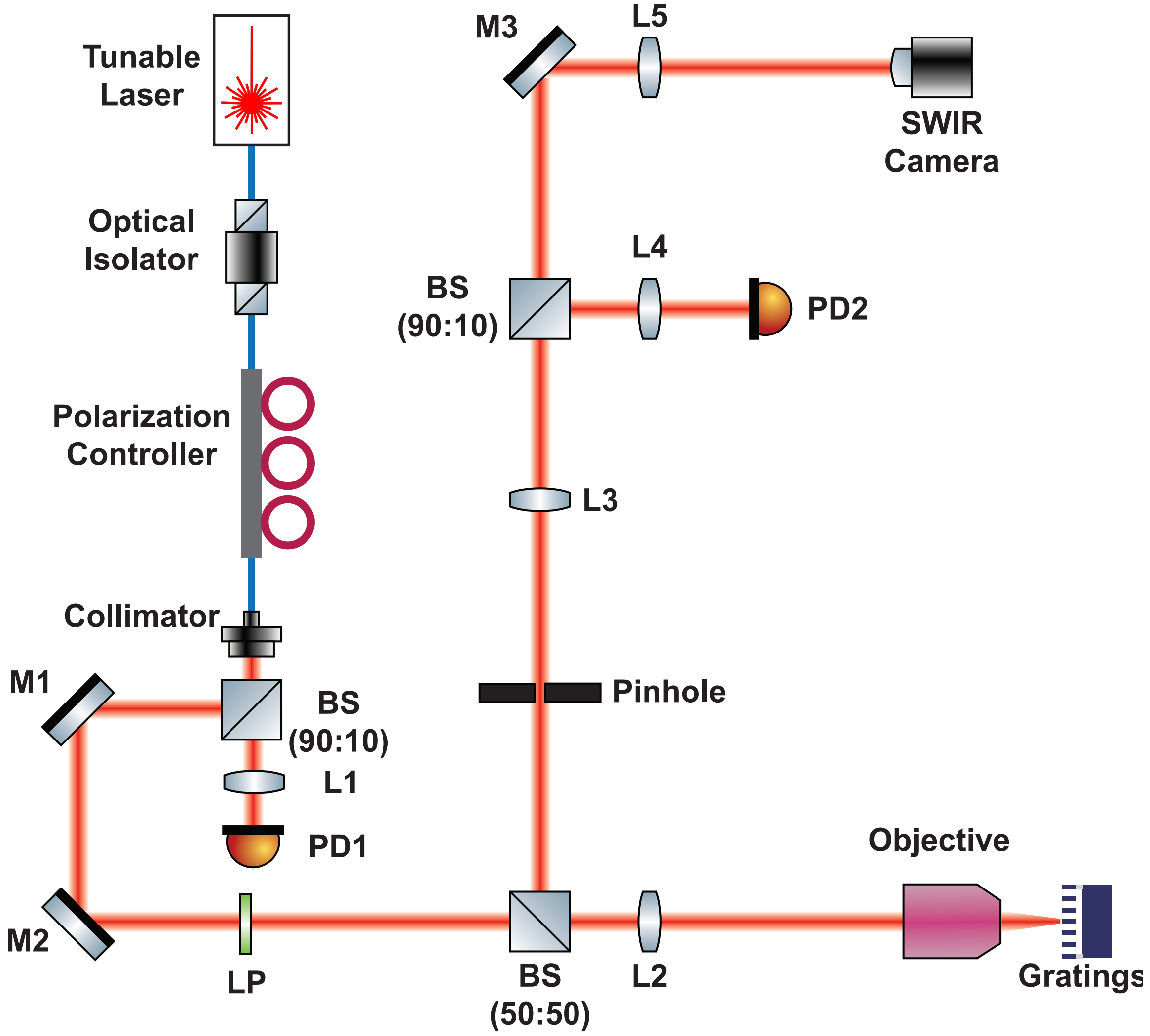


**Figure S7 Schematic illustration of the experimental setup.** Red lines indicate paths of light propagation. LP: linear polarizer. BS: beamsplitter. L: lens. PD: photodetector. M: mirror. Obj.: microscope objective lens. SWIR camera: short-wave infrared camera.

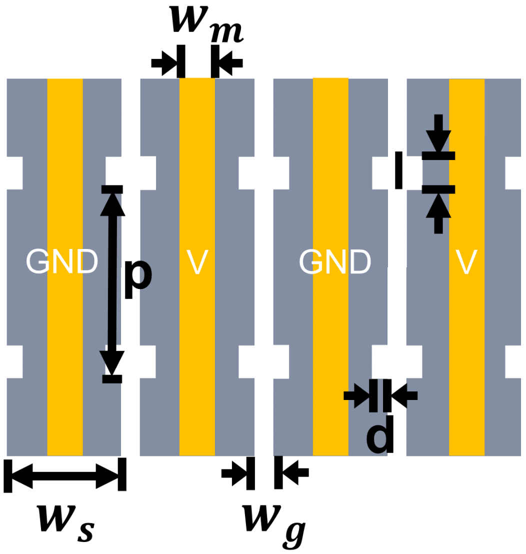


**Figure S8 Label of the device geometry.** p: period of the notch. d: width of the notch. l: length of the notch. $w_s$: slab width. $w_g$: slot width. $w_m$: metal width.

## I. SUPPLEMENTARY REFERENCES


[1] Lawrence, M., Barton III, D. R. & Dionne, J. A. Nonreciprocal flat optics with silicon metasurfaces. *Nano letters* **18**, 1104–1109 (2018).

[2] Lawrence, M. *et al.* High quality factor phase gradient metasurfaces. *Nature Nanotechnology* **15**, 956–961 (2020).

[3] Joannopoulos, J. D., Johnson, S. G., Winn, J. N. & Meade, R. D. Molding the flow of light. *Princeton Univ. Press, Princeton, NJ [ua]* (2008).

[4] Benea-Chelmus, I.-C. *et al.* Electro-optic spatial light modulator from an engineered organic layer. *Nature communications* **12**, 1–10 (2021).

[5] Witmer, J. D. *et al.* A silicon-organic hybrid platform for quantum microwave-to-optical transduction. *Quantum Science and Technology* **5**, 034004 (2020).

[6] Boyd, R. W., Gaeta, A. L. & Giese, E. Nonlinear optics. In *Springer Handbook of Atomic, Molecular, and Optical Physics*, 1097–1110 (Springer, 2008).

[7] Benea-Chelmus, I.-C. *et al.* Gigahertz free-space electro-optic modulators based on mie resonances. *Nature Communications* **13**, 1–9 (2022).

[8] Kwon, H., Zheng, T. & Faraon, A. Nano-electromechanical tuning of dual-mode resonant dielectric metasurfaces for dynamic amplitude and phase modulation. *Nano Letters* **21**, 2817–2823 (2021).
